# ROS-responsive hydrogel loaded with capsaicin promotes tenogenic differentiation of tendon stem/progenitor cells and enhances tendon injury repair

**DOI:** 10.1016/j.mtbio.2025.102707

**Published:** 2025-12-24

**Authors:** Yun-Liang Zhu, Si-Chao Gu, Bao-Liang Lu, Hu Sun, Zai-Yong Guan, Rui-Hua Zhou, Ting-Yong Sun, Wen Gao, Shi-Yuan Fang

**Affiliations:** a Department of Orthopedics, Gaoyou People's Hospital, The Third Clinical Medical College of Yangzhou University, Yangzhou, Jiangsu, 225600, China; b Department of Orthopedics, The First Affiliated Hospital of USTC, Division of Life Sciences and Medicine, University of Science and Technology of China, Hefei, Anhui, China; c The Second Affiliated Hospital of Fuyang Normal University, Fuyang, Anhui, 236001, China

**Keywords:** Capsaicin, Tendon stem/progenitor cells, Biomaterials, Tendon injury, Inflammation

## Abstract

Tendon injury repair remains challenging in sports medicine and orthopedics, partly due to limited endogenous regenerative capacity and sustained low-grade inflammation, which disrupt tendon stem/progenitor cells (TSPCs) homeostasis, impairing tenogenesis and enhancing osteogenesis, leading to complications like heterotopic ossification. Here, we engineered a reactive oxygen species (ROS)-responsive hydrogel loaded with capsaicin (CGT) and explored its role in tendon regeneration. *In vitro*, under IL-1β-induced inflammatory conditions, CGT ameliorated TSPCs dysfunction by upregulating tenogenic markers (Scx, Tnmd, Mkx) and collagen synthesis, while downregulating osteogenic markers (OCN, Runx-2), reducing ALP activity, and inhibiting calcium nodule formation. Mechanistically, CGT may suppress the PI3K-AKT-mTOR axis, attenuating proinflammatory cytokine secretion (IL-6, TNF-α, IL-1β). *In vivo*, local injection of CGT alone or with exogenous TSPCs into SD rat Achilles tendon defects promoted orderly collagen regeneration, reduced inflammation, and inhibited heterotopic ossification at 8 weeks postoperatively, with favorable biosafety. Tissue immunostaining suggested CGT enhanced tenogenesis-related and suppressed osteogenesis-related gene expression. These findings preliminarily indicate that CGT may modulate TSPCs differentiation and inflammatory microenvironments via the PI3K-AKT-mTOR axis, supporting tendon regeneration. This study offers insights into functional biomaterial design, with CGT showing potential for tendon injury management.

## Graphical abstract

**Figure:**
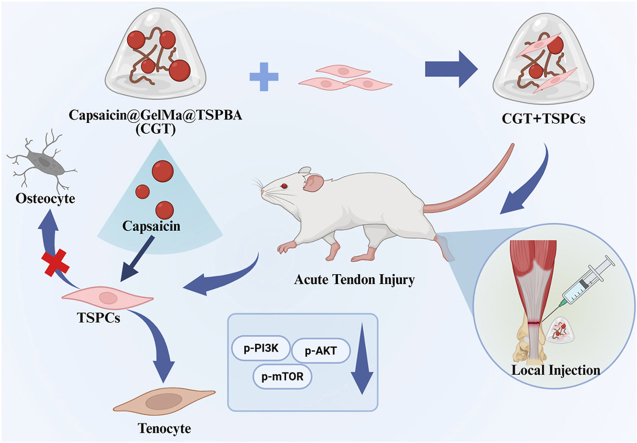

## 1 Introduction

**Scheme 1:**
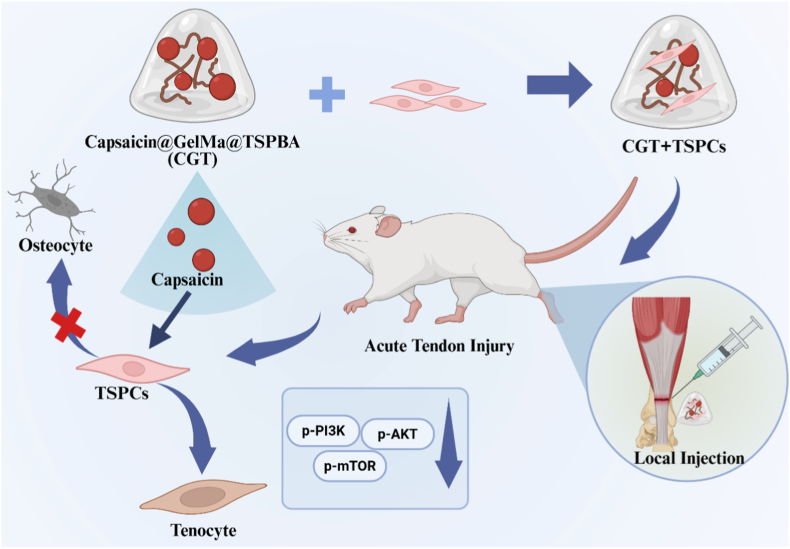
ROS-responsive hydrogel loaded with capsaicin promotes tenogenic differentiation of TSPCs and enhances tendon repair.

As a key connective tissue linking muscles to bones, tendons have limited endogenous repair capacity, making the repair of tendon injuries a significant challenge in the fields of sports medicine and orthopedics [1]. Tendon injury is often accompanied by the activation of local inflammatory responses. Although early inflammation can facilitate the initiation of repair by recruiting stem cells [2], a persistent low-grade inflammatory state leads to dysfunction of tendon stem/progenitor cells (TSPCs), which is specifically manifested as impaired tenogenic differentiation capacity and aberrantly enhanced osteogenic differentiation [3,4]. This further triggers complications such as tendon repair failure and traumatic heterotopic ossification, severely affecting the recovery of patients' motor function. Currently, commonly used clinical repair approaches, such as pharmacological therapy, extracorporeal shock wave therapy [5,6], surgical suture, and autologous/allogeneic tendon transplantation [7,8], are prone to complications including tendon adhesion, heterotopic ossification, poor repair, and post-repair re-rupture, resulting in limited repair efficacy (see Scheme 1).

In response to the limited repair capacity of tendon tissue, stem cell therapy has provided a novel therapeutic strategy for tendon regeneration [9,10]. TSPCs possess cellular functions such as self-renewal, multipotent differentiation, paracrine activity, and immunoregulation [11,12]. TSPCs can regulate tendon tissue homeostasis by differentiating into tenocytes, making them a key cellular candidate for tendon healing [13]. Although exogenous supplementation of TSPCs is an effective strategy for treating tendon injury repair [14], Chen et al. [11] reported that local ectopic cartilage and bone formation occur after *in vivo* administration of exogenous stem cells for tendon repair. This may be attributed to the fact that the inflammatory microenvironment of tendon tissue after injury, especially acute injury, is often overlooked [15], which severely impairs the function of transplanted stem cells and leads to unsatisfactory therapeutic outcomes. Therefore, how to inhibit the local excessive inflammatory response after tendon tissue injury and precisely regulate the differentiation balance of exogenous TSPCs remains a key issue to be addressed.

Hydrogels, as biomaterials with excellent biocompatibility and structural bionic properties, have received extensive attention in tendon tissue engineering [14,16,17]. Gelatin methacrylate (GelMa) has emerged as an ideal carrier for loading cells and bioactive molecules due to its photocurable property, tunable mechanical performance, and superior cytocompatibility. In recent years, the composite strategy of natural active ingredients and functional materials has provided new ideas for improving biomaterial properties. Capsaicin, the main active component of chili peppers, has been confirmed to possess multiple biological functions such as significant anti-inflammatory, analgesic effects, and regulation of cell proliferation and differentiation [18,19]. Recent studies have reported its positive role in tendon injury repair [20], especially its excellent therapeutic effect in relieving pain after tendon tissue injury [21,22]. Sathiamurthi et al. [23] reported that capsaicin can inhibit excessive accumulation of type I collagen, which may provide a potential strategy for preventing postoperative adhesion and fibrosis in tendon injury. However, research on how capsaicin regulates TSPCs functions to promote tendon injury repair is still in the exploratory stage.

Persistent oxidative stress and inflammation are closely associated with poor healing outcomes of adult tendons [24]. Sustained oxidative stress is recognized as a major factor contributing to tendon fibrosis and adhesion following acute tendon injury [24,25]. Reactive oxygen species (ROS) are continuously generated during normal cellular metabolism, and stimuli such as trauma can further enhance their production. Li et al. [26] observed a significant increase in ROS levels in injured patellar tendon tissues compared to normal tendons in a rat model of patellar tendon injury. When ROS are overproduced or cannot be effectively scavenged by the antioxidant system, oxidative stress occurs, which in turn induces cellular dysfunction and tissue damage [25].TSPBA (N1-(4-boronobenzyl)-N3-(4-boronophenyl)-N1,N1,N3,N3-tetramethylpropane-1,3-diaminium) is an ROS-responsive crosslinking agent [24,27]. TSPBA-crosslinked Gelatin Methacryloyl (GelMA-TSPBA) is a functional biomaterial fabricated via crosslinking reaction, where the ROS-responsive crosslinking agent TSPBA is incorporated into the GelMA system [28]. GelMA-TSPBA inherits the excellent biocompatibility, cell adhesion, and degradability of GelMA. Its TSPBA-crosslinked structure enables the formation of a stable three-dimensional hydrogel network, and specific cleavage of crosslinking bonds occurs exclusively in the presence of ROS, allowing for targeted degradation and payload release.

Based on this, the present study aims to construct a functional composite hydrogel loaded with capsaicin (Capsaicin@GelMa@TSPBA, CGT) and systematically investigate its role and mechanism in tendon injury repair. *In vitro* study will use low-concentration IL-1β (10 ng/ml, 3 days) to simulate a persistent inflammatory microenvironment, aiming to explore the regulatory effect of CGT on tenogenic and osteogenic differentiation of TSPCs, with a focus on verifying whether CGT regulates the expression of inflammatory factors by modulating the PI3K-AKT-mTOR signaling axis. In addition, by establishing an SD rat Achilles tendon defect model, the therapeutic effect of CGT alone or in combination with exogenous TSPCs on tendon injury repair will be evaluated *in vivo*. This study is expected to provide experimental evidence for the development of tendon repair biomaterials with anti-inflammatory, tenogenic differentiation-promoting, and heterotopic ossification-inhibiting functions, and offer a potential therapeutic strategy for the precise clinical treatment of tendon injury.

## 2 Materials and methods

*Synthesis of GT (GelMa@TSPBA) and CGT*: First, 2g of GelMA powder (Engineering for Life, China) were precisely measured and dissolved in 50 ml of deionized water. The resulting mixture was subjected to magnetic stirring within a constant-temperature water bath maintained at 45 °C for a duration of 30–40 min, until a homogeneous and transparent solution was achieved. To prevent gelation due to temperature reduction, this solution was kept warm for subsequent use. Seconrd, 1g EDC, 0.5g NHS, and 1g TSPBA were sequentially weighed. These reagents were introduced into a mixed solvent composed of 50 ml of deionized water and 15 ml of dimethyl sulfoxide (DMSO) in sequence. The resulting mixture was stirred at ambient temperature for 15 min until complete dissolution was achieved, resulting in a clear activated mixed solution, with TSPBA exhibiting excellent water solubility due to the presence of sulfonic acid groups. Then, the activated mixed solution was gradually added dropwise to the pre-warmed GelMA aqueous solutions of varying concentrations. The mixture was stirred at 45 °C at a speed of 500 r/min to ensure uniform mixing. Following the adjustment of the mixture's pH to 5.5, the solution was subjected to continuous stirring at ambient temperature for a duration of three days to facilitate covalent coupling between the carboxyl groups of GelMA, present at final concentrations of 5 % (w/v), 10 % (w/v), and 15 % (w/v), and the amino groups of TSPBA. Upon completion of the reaction, the resultant solution underwent dialysis against deionized water for three days. Subsequently, the dialyzed solution was subjected to freeze-drying, resulting in the formation of a white, loose GelMA-TSPBA powder. This powder was then sealed and stored at 4 °C for future use. The preparation of the CGT, 100 mg of capsaicin was dissolved in 10 ml of ethanol to create a stock solution. Subsequently, a 10 % (w/v) GelMA solution was prepared by dissolving it in a water bath maintained at 60 °C, followed by centrifugation at 5000 rpm to eliminate air bubbles. Concurrently, an equivalent volume of TSPBA solution was prepared and subjected to identical centrifugation conditions to ensure bubble removal, achieving a total concentration of 10 % upon mixing. The capsaicin stock solution was then incorporated into the GelMA solution to attain a final concentration of 10 μM, ensuring thorough mixing. This blend was subsequently combined with the TSPBA solution in a 1:1 ratio and subjected to reciprocal extrusion 5–8 times using a 1 ml syringe. The resultant mixture underwent crosslinking under a 405 nm light source for 30 s to form the hydrogel. Finally, unreacted components were eliminated through water washing, and the product was freeze-dried to yield the CGT composite hydrogel. All prepared solutions were sterilized via pasteurization. The specific procedure was as follows: the solutions were heated to 80 °C and maintained at this temperature for 20 min, then quickly transferred to an ice-water mixture for 5 min of immersion. This aforementioned operation was repeated once to complete the sterilization process. For characterization, GT samples were lyophilized into powder and examined with SEM, while a rheometer assessed the hydrogels' rheological properties and swelling behavior. CGT samples, shaped rectangularly, were immersed in PBS and 5 % H_2_O_2_, incubated at 37 °C, and sampled on days 1, 3, 5, 7, 14, 21, and 28. Capsaicin release was measured using UV–Vis spectrophotometry to construct a drug release profile.

*Isolation and Culture of TSPCs*: For the isolation of TSPCs [12,29], Achilles tendon tissues were obtained from 8-week-old male Sprague-Dawley rats. The procedure was as follows: initially, the rats were anesthetized via intraperitoneal injection, immobilized, and the surgical site was depilated and disinfected to ensure full exposure of the bilateral Achilles tendons. Subsequently, the Achilles tendons were carefully isolated, with the surrounding soft tissues and tendon sheaths meticulously removed. The middle segment of the tendon tissue was excised and rinsed three times with pre-cooled phosphate-buffered saline (PBS). The tissue was then sectioned into small pieces within a biosafety cabinet and transferred to a complete medium containing 3 mg/ml type I collagenase for digestion. The complete medium comprised low-glucose Dulbecco's modified Eagle's medium (LG-DMEM), supplemented with 100 U/ml penicillin, 100 mg/ml streptomycin, and 10 % fetal bovine serum (FBS). The centrifuge tube containing the tissue was placed in a cell incubator and gently agitated every 30 min to facilitate digestion. Following complete digestion of the tissue into a transparent suspension, the mixture was gently pipetted to achieve homogenization and subsequently filtered through a 70 μm cell strainer to obtain a single-cell suspension. This suspension was then subjected to centrifugation at 1000 rpm for 5 min. Post-centrifugation, the supernatant was discarded, and the cells were resuspended in fresh complete medium before being seeded into culture flasks. These flasks were incubated in a cell incubator set at 37 °C with 5 % CO_2_. Once the cells reached a confluency of 80 %–90 %, they were digested with trypsin and pooled as passage 0 (P0) cells. Cells from passages 1 to 3 (P1–P3) were subsequently selected for further experimentation.

*Immunofluorescence*: Immunofluorescence involved culturing cells, fixing them with 4 % paraformaldehyde, and washing with PBS. Cells were permeabilized with 0.1 % Triton X-100 and blocked with 5 % BSA to minimize nonspecific binding. After washing, cells were incubated with the primary antibody for 12 h at 4 °C, washed again, and then incubated with a fluorescently labeled secondary antibody for 2 h at 37 °C. Following a final wash, fluorescent signals were observed and recorded using a fluorescence microscope to assess the target protein's expression and distribution. Comprehensive details regarding the antibodies used are available in Supplementary Table 2.

*Tenogenic Differentiation*: Tendon stem/progenitor cells (TSPCs) were initially seeded into 6-well plates and cultured until they reached approximately 80 % confluence. Subsequently, the cells were exposed to a tenogenic induction medium, consisting of high-glucose Dulbecco's Modified Eagle Medium (DMEM) supplemented with 50 μg/ml ascorbic acid, 10 % fetal bovine serum (FBS), and 1 % penicillin-streptomycin, to promote tenogenic differentiation. The culture medium was routinely refreshed throughout the induction period to maintain optimal conditions. On the 14th day of induction, Sirius Red (SRE) staining (Solarbio, China) was conducted to assess the extent of differentiation.

*Osteogenic Differentiation*: TSPCs were cultured in 6-well plates and co-cultured with CGT. The standard medium was substituted with an osteogenic induction medium comprising 10 % fetal bovine serum (FBS), 1 % penicillin-streptomycin (PS), 10 mM β-glycerophosphate, 50 μM L-ascorbic acid, and 100 nM dexamethasone. Following a 14-day incubation period, an alkaline phosphatase assay was conducted, with absorbance measured by determining the optical density at 520 nm. To assess extracellular matrix mineralization, Alizarin Red S (ARS) staining was performed. After 21 days of incubation, ARS staining was carried out, and observations were made using a Leica microscope. The ARS complexes were subsequently dissolved in a cetylpyridinium chloride solution, and absorbance was quantitatively measured via spectrophotometric analysis at 562 nm using a microplate reader.

*Western Blot*: Proteins were extracted from cell samples, and their concentrations were quantified utilizing the bicinchoninic acid (BCA) assay. Following this, the samples underwent thermal treatment at 100 °C for 15 min, and the proteins were subsequently resolved via sodium dodecyl sulfate-polyacrylamide gel electrophoresis (SDS-PAGE). The resolved proteins were then transferred onto polyvinylidene fluoride (PVDF) or nitrocellulose membranes, which were blocked using 5 % skimmed milk powder or bovine serum albumin (BSA). The membranes were incubated with the appropriate primary antibodies at 4 °C for a duration of 12 h, followed by a series of washes with a wash buffer. On the subsequent day, enzyme-conjugated secondary antibodies were introduced and incubated at 37 °C for 2 h, after which the membranes underwent additional washing. The expression levels of the target proteins were analyzed relative to those of internal reference proteins. Comprehensive details regarding the antibodies used are available in Supplementary Table 1.

*qPCR*: RNA was isolated from the samples utilizing a commercial kit (Beyotime, China), and complementary DNA (cDNA) synthesis was performed via reverse transcriptase. The reaction mixture was subsequently transferred to a quantitative PCR (qPCR) instrument, employing SYBR Green-based detection, in conjunction with a PCR buffer (Beyotime, China). An appropriate amplification cycling program (an initial denaturation step at 95 °C for 10 min, followed by 40 amplification cycles. Each cycle comprised a denaturation phase at 95 °C for 15 s and an annealing/extension phase at 60 °C for 60 s. A final melting curve analysis was performed by incrementally increasing the temperature from 60 °C to 95 °C at a rate of 0.3 °C every 15 s to confirm the specificity of the amplified products) was established. Fluorescent signals were monitored in real-time to quantify the expression levels of the target genes. The relative expression levels were determined by comparing the cycle threshold (Ct) values of the target genes with those of internal reference genes within the samples, followed by statistical analysis. Comprehensive details of each primer are provided in Supplementary Table 3.

*RNA-seq:* Total RNA was first isolated from samples using TRIzol® Reagent (Invitrogen, USA); RNA purity and integrity were then assessed separately via NanoDrop 2000 (Thermo Fisher Scientific, USA) and Agilent 2100 Bioanalyzer (Agilent Technologies, USA), with an RNA integrity number ≥7.0 required for subsequent analysis. Next, strand-specific cDNA libraries were constructed using the NEBNext® Ultra™ RNA Library Prep Kit for Illumina® (NEB, USA) in accordance with the manufacturer's instructions. Finally, the constructed libraries were sequenced on an Illumina NovaSeq 6000 platform (Illumina, USA) to generate 150 bp paired-end reads; raw reads were filtered using Trimmomatic (v0.39) to remove low-quality reads and adaptor sequences, clean reads were mapped to the via HISAT2 (v2.2.1), and differentially expressed genes (DEGs) were identified using DESeq2 (v1.34.0) with the screening criteria of |log2(fold change)| ≥ log2(2) and adjusted P-value <0.05.

*Tendon Defect Model*: In this study, 8-week-old specific pathogen-free (SPF) male Sprague-Dawley (SD) rats, weighing 220–250 g, were used. The rats were housed in a barrier environment with a constant temperature of 25 °C and a 12-h light/12-h dark cycle, and had free access to standard chow and sterile water. To reduce anesthesia-related risks, food and water were withheld from the rats for 6 h before surgery. After surgery, the surgical incisions were disinfected with povidone-iodine twice daily, and the rats were housed individually until the incisions healed. During this period, the activity of rats and their feeding status were closely monitored. Experimental grouping was performed via complete randomization using a random number table. All sample detection and data statistics were conducted by researchers who were unaware of the treatment groups (i.e., blinding was implemented). Anesthesia was administered via intraperitoneal injection of 1 % pentobarbital. During the surgery, the fascia anterior to the Achilles tendon was first bluntly incised, followed by release of the Achilles tendon. A full-thickness standard defect (2 mm in radius) [30] was created in the middle segment of the Achilles tendon (8 mm from the calcaneal tuberosity) using a biopsy puncture needle. Postoperatively, 0.5 % buprenorphine (0.1 mg/kg body weight) was administered via intraperitoneal injection twice daily for 3 consecutive days. A uniform volume of 50 μL hydrogel was injected into each rat during surgery. The rats were divided into three groups based on the type of hydrogel injected into the defect: the GT group (GelMa@TSPBA), the CGT group (Capsaicin@GelMa@TSPBA), and the CGT + TSPCs (Capsaicin@GelMa@TSPBA + TSPCs) group (5 SD rats per group). Samples were collected 8 weeks after surgery for subsequent experiments.

*Combination of CGT and TSPCs*: Prior to *in vivo* injection, TSPCs in the logarithmic growth phase were digested using 0.25 % trypsin, followed by centrifugation at 1000 r/min for 5 min, after which the supernatant was discarded. The cells were then resuspended in sterile phosphate-buffered saline (PBS). This cell suspension was subsequently mixed thoroughly with CGT hydrogel, pre-warmed to 37 °C to maintain its fluid state, at a volume ratio of 1:1, resulting in a TSPCs-CGT composite system with a final cell concentration of 1 × 10^6^ cells/mL [14]. This composite system was then exposed to a 405 nm light source for 30 s. Immediately following this, a 1 ml sterile syringe was employed to inject the composite system into the Achilles tendon injury site of rats.

*Statistical analyses*: Prior to conducting parametric tests, such as the two-independent-sample *t*-test and one-way analysis of variance (ANOVA), we assessed the normality of all datasets using the Shapiro-Wilk test. These parametric tests were employed only after confirming that the data adhered to a normal distribution. For determining the appropriate sample size, we conducted a power analysis using G*Power 3.1 software, setting the significance level (α) at 0.05, the power (1-β) at 0.8, and the effect size at 0.8, based on findings from preliminary experiments. Consequently, the final sample size was established as 5 rats per group for animal experiments and 3 independent replicates for cell experiments, ensuring adequate power for statistical analyses. All experimental replicates reported in this study are biological replicates, meaning each replicate involved independently prepared samples, such as TSPCs from different culture batches for cell experiments and samples from individual rats for animal experiments, rather than technical replicates.

## 3 Result

### 3.1 Characterization of CGT

**Fig. 1:**
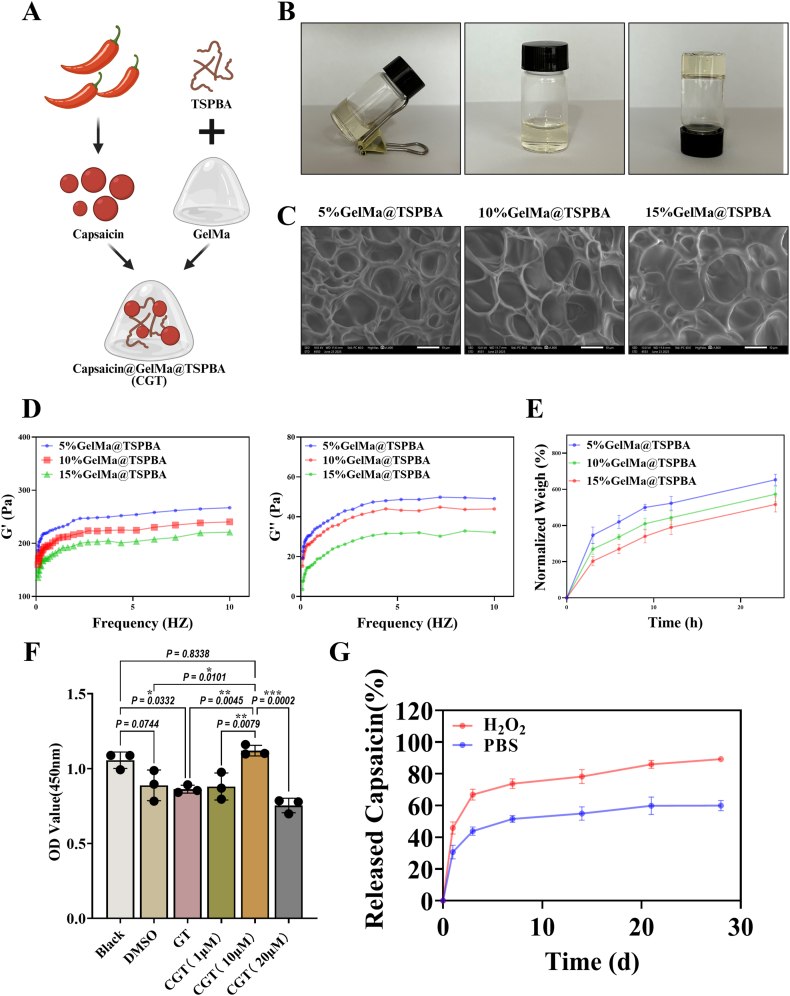
**Characterization of GT and CGT.** (A)Schematic of CGT synthesis process. (B)CGT hydrogel morphology. (C)SEM images of GT added at different concentrations (5 %, 10 %, 15 %) of GelMa. Scale bar, 10 μm. (D)G′ and G″ of GT hydrogels under 37 °C. (E)Swelling ratio of GT with different concentrations (5 %, 10 %, 15 %) of GelMa. (F)Evaluation of proliferation of TSPCs treated with 1 μM, 10 μM, and 20 μM capsaicin for 3 days. (G) Comparison of capsaicin release efficiency in PBS and 5 % H_2_O_2_ solutions versus the 10 μM standard solution. *P < 0.05, **P < 0.01, ***P < 0.001.

The schematic diagram of CGT synthesis is shown in Fig. 1A. We first observed the morphology of the hydrogels from different perspectives (Fig. 1B) and investigated the mixtures of GelMa at different concentrations (5 %, 10 %, 15 %) with TSPBA using scanning electron microscopy (SEM). As shown in Fig. 1C, well-defined porous structures were observed in all mixtures of GelMa at different concentrations with TSPBA, and the porosity gradually decreased with increasing GelMa concentration. Porosity plays a crucial role in the process of nutrient exchange. Fig. 1D illustrates the correlation between G’ (storage modulus) and G’’ (loss modulus) with GelMa content. The G′ and G″ values of GT(GelMa@TSPBA) with different GelMa ratios exhibited variations within the analyzed frequency range at 37 °C. Furthermore, the results showed that the G′ values of GT with different GelMa concentrations were all greater than G″, indicating that GT possesses strong mechanical stability. The ability of hydrogel materials to absorb and retain liquids is a key determinant of their potential applications in biomedicine. The swelling behavior of GT hydrogels was evaluated at 37 °C (Fig. 1E). The highest swelling degree was observed for the GT hydrogel with 10 % GelMa. The results demonstrated that the swelling degree of GT hydrogels showed a significant decrease with increasing GelMa content. We finally selected 10 % GelMa concentration to mix with TSPBA. To determine the optimal concentration of capsaicin, we used the CCK-8 assay to compare the effects of different capsaicin concentrations on the proliferation ability of TSPCs. Wang et al. [31] reported that 1 μM capsaicin could promote the proliferation of periodontal ligament stem cells, while Ibrahim Muhammed [32] reported that 10 μM capsaicin could promote ROS production in BMSCs and inhibit proliferation, with stronger cytotoxicity at higher concentrations. Based on this, we mixed GT with capsaicin at concentrations of 1 μM, 10 μM, and 20 μM. The results showed that CGT formed by mixing 10 μM capsaicin with GT promoted the proliferation of TSPCs for 3 days (Fig. 1F). We further tested CGT loaded with 10 μM capsaicin in PBS and H_2_O_2_ respectively. The results showed that the cumulative release amount reached nearly 80 % on day 14 and exceeded 85 % on day 21 in H_2_O_2_, whereas it only reached 60 % on day 28 in PBS (Fig. 1G). These findings verify the ROS responsiveness of CGT, enabling it to achieve targeted controlled release in regions with high oxidative stress.

### 3.2 CGT promotes tenogenic differentiation of tendon stem/progenitor cells *In vitro*

**Fig. 2:**
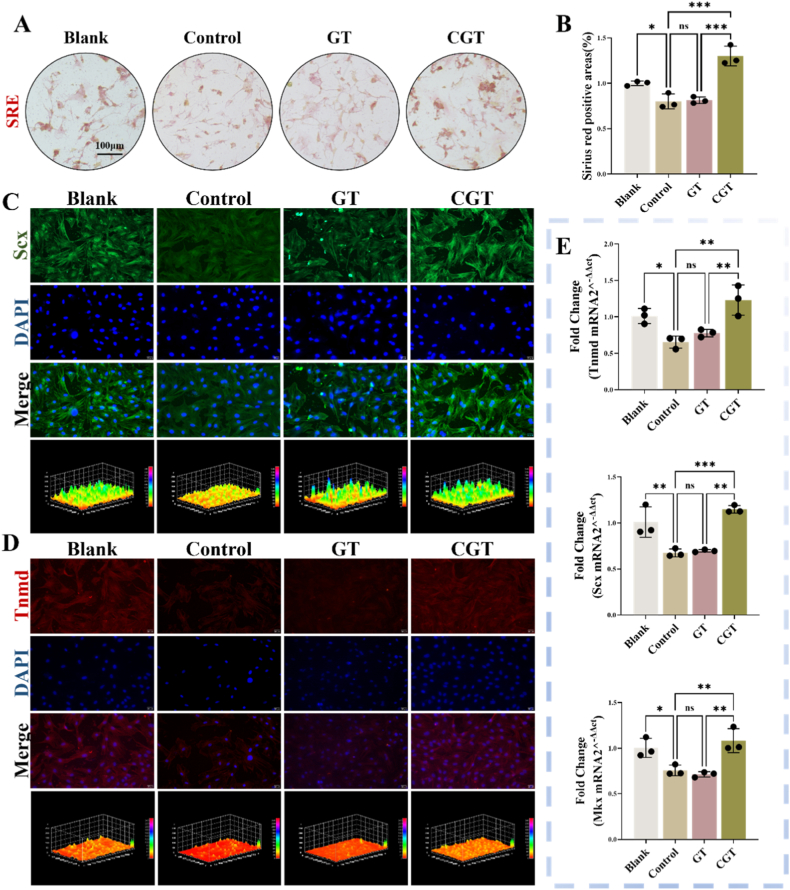
**CGT promotes tenogenic differentiation of TSPCs *in vitro.*** (A)SRE staining of TSPCs cultured with IL-1β, GT or CGT for 14 days, Scale bar: 100 μm (B)Quantitative analysis of SRE staining. (C)Immunofluorescence staining of tenogenesis-related gene Scx and (D) Tnmd in TSPCs cultured for 7 days, Scale bar: 30 μm (E)qPCR analysis of the relative changes in mRNA expression level of tenogenesis-related genes (Tnmd, Scx, Mkx). *P < 0.05, **P < 0.01, ***P < 0.001,****P < 0.0001.

In the early stage of tendon injury, inflammatory factors are released to recruit stem cells; however, the persistence of low-grade inflammation may lead to dysfunction of tendon stem cells, which in turn can result in secondary tendon repair failure. We used 10 ng/ml IL-1β to intervene in TSPCs to simulate the effect of persistent low-grade inflammation on recruited TSPCs during tendon injury repair. To further verify the role of CGT on TSPCs, we divided TSPCs into four groups based on different intervention methods: the Blank group, the Control group (IL-1β), the GT group (IL-1β+GelMa@TSPBA), and the CGT group (IL-1β+Capsaicin@GelMa@TSPBA), with all interventions lasting for 7 days. Sirius Red staining can specifically visualize collagen fibers. The results of Sirius Red staining showed that compared with the Blank group, IL-1β intervention reduced the *in vitro* collagen secretion of TSPCs (Fig. 2A–B), while after the addition of CGT, the area of *in vitro* collagen formation in TSPCs was significantly larger than that in the Control group. We further detected the expression of tenogenic markers (Scx, Tnmd) in each group using cellular immunofluorescence. As shown in Fig. 2C–D, compared with the Blank group, IL-1β intervention decreased the expression of Scx and Tnmd in TSPCs, and CGT intervention could partially alleviate the inhibitory effect of IL-1β on the expression of tenogenic markers Scx and Tnmd in TSPCs. Meanwhile, we used qPCR to detect the changes in mRNA expression levels of tenogenesis-related genes Tnmd, Scx, and Mkx in TSPCs of each group. The results showed that compared with the Blank group, IL-1β intervention decreased the mRNA expression of Tnmd, Scx, and Mkx in TSPCs, while the addition of CGT could partially increase the mRNA expression of Tnmd, Scx, and Mkx in TSPCs (Fig. 2E). These results indicate that persistent low-grade inflammation can reduce the tenogenic differentiation capacity of TSPCs, while CGT can partially alleviate the inhibitory effect of IL-1β on the tenogenic differentiation capacity of TSPCs.

### 3.3 CGT inhibits osteogenic differentiation of tendon stem/progenitor cells *In vitro*

**Fig. 3:**
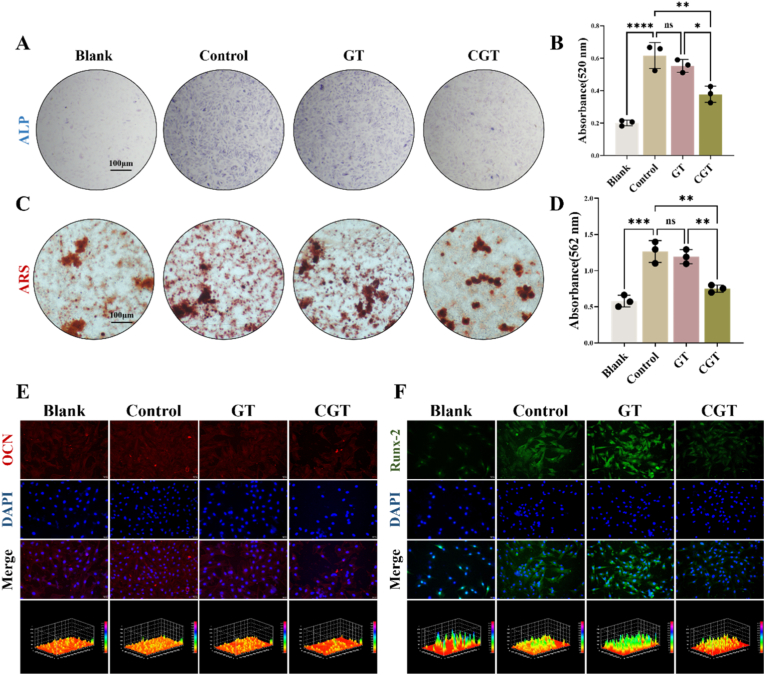
**CGT inhibits osteogenic differentiation of TSPCs *in vitro.*** (A)ALP staining of TSPCs cultured with IL-1β, GT or CGT for 14 days, Scale bar: 100 μm. (B)Quantitative analysis of ALP staining. (C)ARS staining of TSPCs cultured with IL-1β, GT or CGT for 21 days, Scale bar: 100 μm. (D)Quantitative analysis of ARS staining. (E)Immunofluorescence staining of osteogenesis-related gene OCN and (F) Runx-2 in TSPCs cultured for 7 days, Scale bar: 30 μm *P < 0.05, **P < 0.01, ***P < 0.001,****P < 0.0001.

The occurrence of persistent low-grade inflammation after tendon injury is closely associated with the development of traumatic heterotopic ossification in tendons, which may be related to the differentiation imbalance of tendon stem cells. The dysfunctional differentiation of TSPCs is a key factor contributing to the difficulty and failure of tendon repair, which can further lead to the development of tendinopathy. We further evaluated the osteogenic differentiation capacity of TSPCs in each group. Alkaline phosphatase (ALP) staining and Alizarin Red staining were used to assess the early and late osteogenic potential of TSPCs, respectively. ALP staining results showed that compared with the Blank group, 10 ng/ml IL-1β intervention enhanced ALP staining intensity, while the addition of CGT partially reduced the ALP staining signal in TSPCs (Fig. 3A–B). Similarly, as shown in Fig. 3C–D, the addition of 10 ng/ml IL-1β to TSPCs increased the staining of calcium nodules, whereas the introduction of CGT partially decreased the capacity of TSPCs to form calcium nodules. These findings indicate that CGT can partially attenuate the promoting effect of IL-1β on both early and late osteogenic differentiation of TSPCs. In addition, we detected the protein expression of osteogenesis-related genes osteocalcin (OCN) and Runx-2 in TSPCs of each group using cellular immunofluorescence. The immunofluorescence results (Fig. 3E–F) showed that compared with the Blank group, the expression of OCN and Runx-2 was upregulated in the Control group, while CGT intervention partially alleviated the IL-1β-induced enhancement of osteogenesis-related gene expression in TSPCs. Collectively, these results demonstrate that *in vitro* treatment with 10 ng/ml IL-1β can enhance the osteogenic differentiation capacity of TSPCs, whereas CGT can partially inhibit the osteogenic differentiation of TSPCs.

### 3.4 CGT promotes TSPCs functions by regulating the expression of cellular proinflammatory factors

**Fig. 4:**
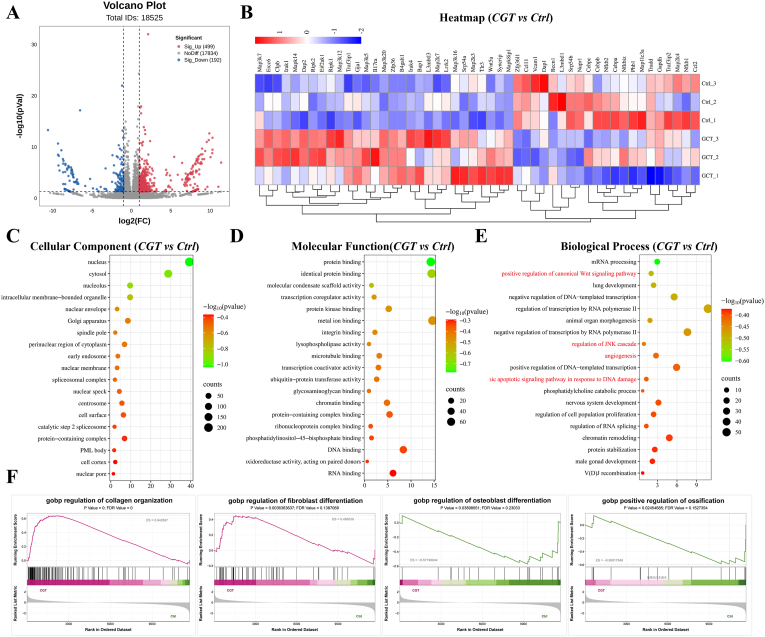
**Analysis of transcriptomic results of TSPCs in the control and CGT-treated Group.** (A)Volcano plot of gene expression of upregulated (499) and downregulated (192) genes (|log_2_(Fold Change)| > log_2_(2) (equivalent to a 2-fold change) and p-value <0.05) in TSPCs after CGT treatment. (B)Gene heatmap of expression differences in inflammation-related genes. (C)The significantly enriched CC terms, (D)MF terms, and (E)BP terms from all DEPs (Ctrl vs. CGT, *P* < 0.05). (F)GSEA plots associated with regulation of collagen organization, fibroblast differentiation, osteoblast differentiation, and ossification.

To further explore the mechanism by which CGT enhances the tenogenic differentiation capacity of TSPCs, we performed mRNA transcriptome sequencing to evaluate the effect of CGT on IL-1β-intervened TSPCs. The volcano plot showed the differences in gene expression in the CGT group compared with the Control group, with a total of 691 genes exhibiting significant changes by more than 2-fold. Among these, 499 genes were upregulated and 192 genes were downregulated. The differential expression of inflammation-related genes is presented in the heatmap (Fig. 4B). We conducted Gene Ontology (GO) enrichment analysis of the differentially expressed genes; the results related to Cellular Component and Molecular Function are shown in Fig. 4C–D. In addition, the results of biological processes (BP) indicated that the mechanism of CGT intervention may be associated with processes in TSPCs such as positive regulation of canonical Wnt signaling pathway, regulation of JNK cascade, angiogenesis, intrinsic apoptotic signaling pathway in response to DNA damage, etc. (Fig. 4E). Gene Set Enrichment Analysis (GSEA) results showed that after CGT intervention, the process of ECM remodeling (Extracellular matrix structural consituent) was upregulated (Fig. S1A), with angiogenesis involved in wound healing (Fig. S1B) and immune regulation, such as positive regulation of adaptive immune response and regulation of adaptive immune response (Fig. S1C–D) being downregulated. Additionally, tenogenesis-related processes, including regulation of collagen organization and regulation of fibroblast differentiation were upregulated, while osteogenesis-related processes such as regulation of osteoblast differentiation and regulation of ossification were downregulated (Fig. 4F).

**Fig. 5:**
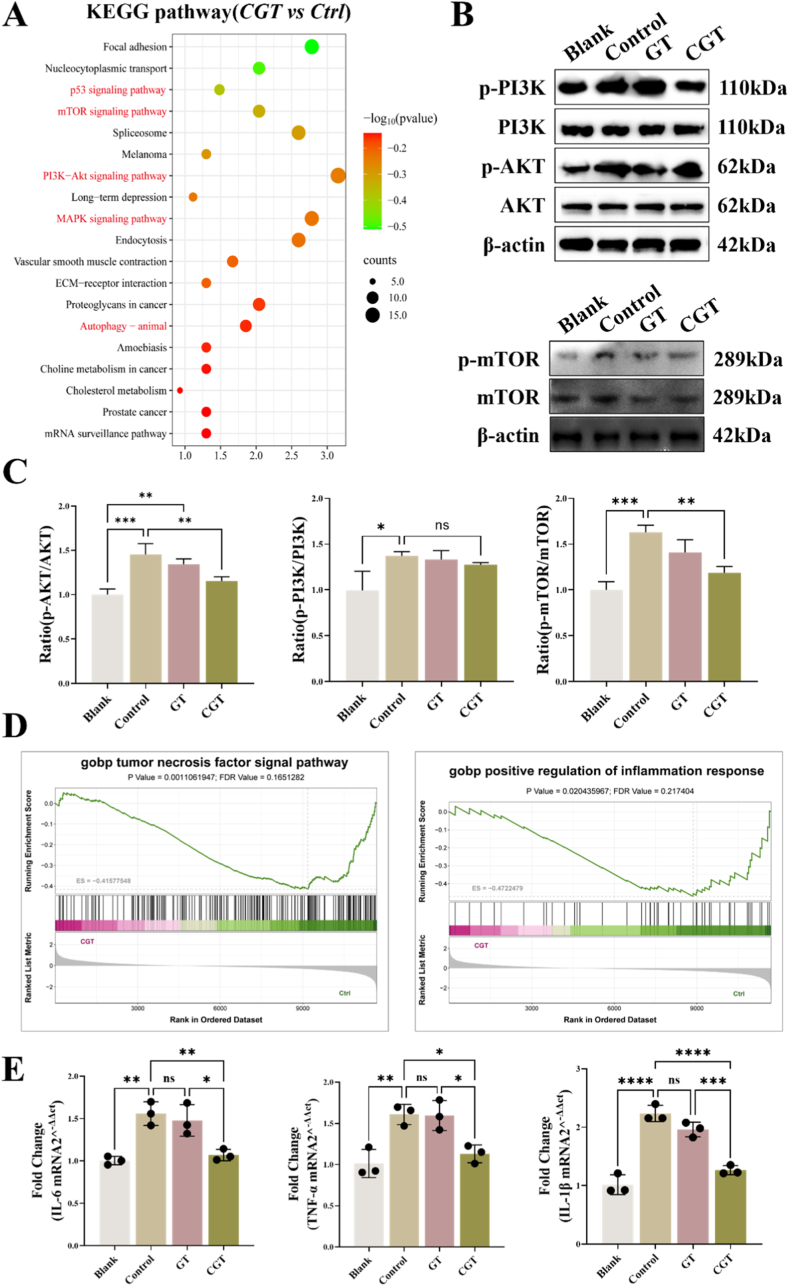
**CGT promotes TSPCs functions by regulating the expression of cellular inflammatory factors.** (A)Top 20 KEGG (Kyoto Encyclopedia of Genes and Genome) enrichment pathway. (B)Western Blot of the changes in the protein expression level of p-PI3K, PI3K, p-AKT, AKT, p-mTOR, and mTOR in TSPCs and (C)semiquantitative analysis. (D)GSEA plots associated with tumor necrosis factor signaling pathway and positive regulation of inflammatory response. (E) qPCR analysis of the relative changes in mRNA expression level of inflammation-related genes (IL-6, TNF-α, IL-1β). *P < 0.05, **P < 0.01, ***P < 0.001,****P < 0.0001.

Kyoto Encyclopedia of Genes and Genomes (KEGG) enrichment analysis revealed that compared with the Control group, CGT intervention significantly altered pathways such as the p53 signaling pathway, mTOR signaling pathway, PI3K-AKT signaling pathway, MAPK signaling pathway, endocytosis, and autophagy (Fig. 5A). We further detected changes in the expression of molecules related to the PI3K-AKT-mTOR signaling axis in cells from each group using Western Blot (as shown in Fig. 5B). Further semi-quantitative analysis revealed that compared with the Blank group, IL-1β intervention in TSPCs increased the expression of p-PI3K, p-AKT, and p-mTOR, while CGT intervention decreased the expression of p-AKT and p-mTOR in TSPCs (Fig. 5C). In addition, we employed the PI3K-AKT phosphorylation agonist 740 Y-P to intervene in TSPCs that were either exposed to the composite CGT or untreated. The result of WB showed that treatment with 740 Y-P partially counteracted the effects of CGT on TSPCs, notably by mitigating the reduction in PI3K-AKT phosphorylation levels and the suppression of IL-1β overexpression (Fig. S2A). Similarly, administration of MHY 1458, an mTOR phosphorylation agonist, diminished the impact of CGT on mTOR phosphorylation levels and IL-1β expression in TSPCs (Fig. S2B). These results suggest that CGT may exert its effects by reducing the phosphorylation levels of AKT and mTOR in the PI3K-AKT-mTOR signaling axis. Notably, further KEGG analysis revealed that CGT intervention significantly downregulated inflammation-related processes, including the tumor necrosis factor signaling pathway and positive regulation of inflammatory response (Fig. 5D). We detected changes in the mRNA levels of inflammation-related genes (IL-6, TNF-α, and IL-1β) in cells from each group using qPCR. The results showed that compared with the Blank group, IL-1β intervention increased the mRNA levels of IL-6, TNF-α, and IL-1β, while CGT could partially inhibit the expression of these inflammatory factors in TSPCs (Fig. 5E). Furthermore, fluorescence results showed that after TSPCs were treated with composite CGT, the intracellular ROS levels decreased. However, treatment with 740 Y-P attenuated the inhibitory effect of CGT on excessive ROS expression in TSPCs (Fig. S3). Collectively, these findings indicate that CGT may promote tenogenic differentiation of TSPCs by reducing the secretion of inflammatory factors in TSPCs through the PI3K-AKT-mTOR signaling axis.

### 3.5 CGT and CGT loaded with exogenous TSPCs promote Achilles tendon injury repair

**Fig. 6:**
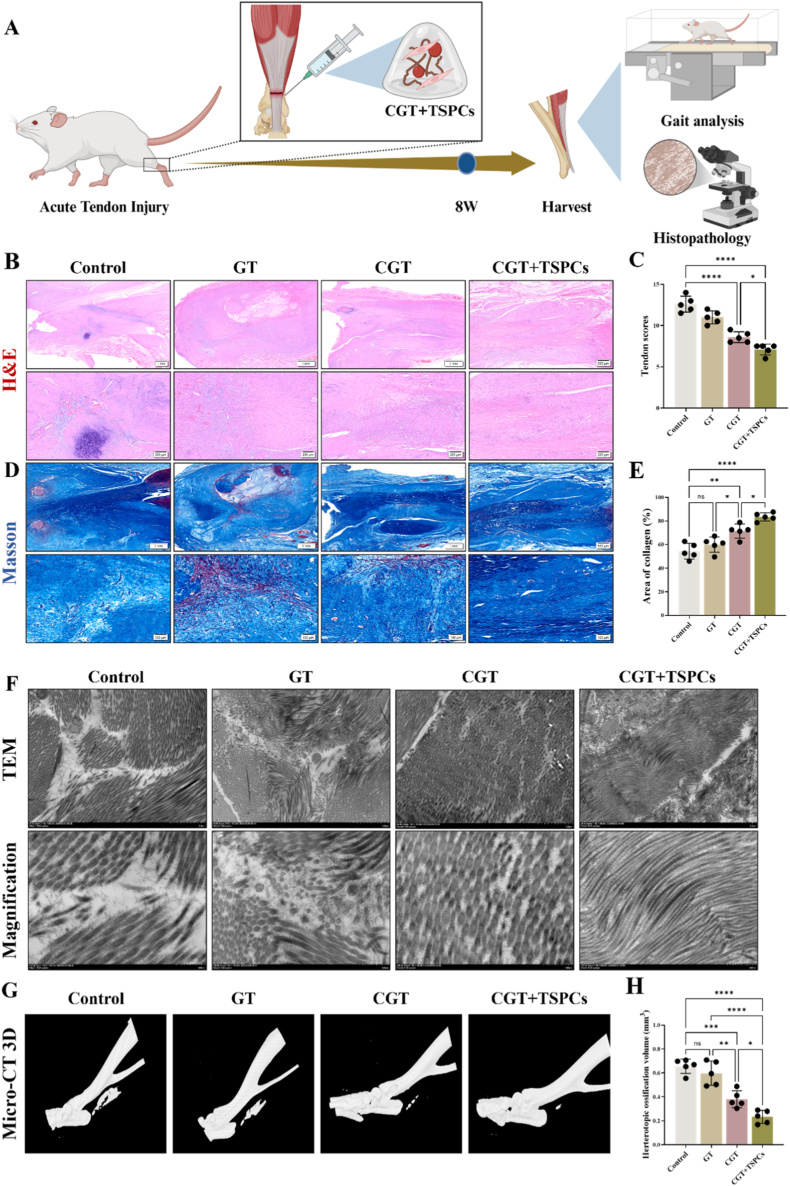
**CGT and CGT loaded with exogenous TSPCs promote Achilles tendon injury repair.** 20 SD rats were subjected to Achilles tendon defect modeling and randomized into four groups: Control, GT, CGT, and CGT + TSPCs (n = 5 per group). The rats were sacrificed at 8 weeks postoperatively for histological evaluation. (A)Scheme of experimental plan *in vivo***.** (B)H&E staining and (C)tendon tissue scoring. (D)Masson staining and (E)semiquantitative analysis of the collagen area. (F)TEM images of tendon tissue, Scale bar: 2 μm and 500 nm (magnification). (G) Micro-CT 3D reconstruction of the SD rats Achilles tendon at week 8, and (H)bone volume quantification of local heterotopic ossification in micro-CT. *P < 0.05, **P < 0.01, ***P < 0.001,****P < 0.0001.

All animal experiments were approved by the Ethics Committee of Anhui Medical University. To better evaluate the *in vivo* effect of CGT on Achilles tendon injury repair in SD rats, we locally injected hydrogels of each group into the Achilles tendon defect site of rats. In the CGT + TSPCs group, TSPCs were derived from the Achilles tendons of 8-week-old adult SD rats(Fig. 6A). Initially, we assessed the *in vivo* biodistribution, retention time, and dynamic metabolism of GT@Cy5 through *in vivo* fluorescence imaging. At one day post-operation, the fluorescent signal of GT@Cy5 in the affected limb of rats was relatively weak. However, by 3 days, the signal was significantly enhanced and concentrated in the affected limb area. This elevated level was sustained at 7 days and gradually diminished by 14 days (Fig. S4). At 8 weeks postoperatively, Achilles tendons and major organs from each group were collected for pathological evaluation. We first verified the biosafety of hydrogels in each group by performing histological assessment of heart, liver, spleen, lung, and kidney tissues (Fig. S5), and no obvious damage was observed in vital organs after hydrogel implantation in any group. Hematoxylin and eosin (HE) staining results of Achilles tendon tissues at 8 weeks postoperatively showed no large-area defects in all groups. In the Control group and GT group, obvious vascular formation and inflammatory cell infiltration were observed locally, with disordered arrangement of local cells and collagen. Compared with the Control group, no obvious angiogenesis was observed in the CGT group and CGT + TSPCs group; however, slight disorder in cell and collagen arrangement was still noted in the CGT group, while the arrangement of newly formed collagen at the injury site in the CGT + TSPCs group was superior to that in other groups (Fig. 6B). Based on semi-quantitative analysis, the tendon tissue scores in the CGT group and CGT + TSPCs group were significantly lower than those in the Control group, indicating better tendon healing outcomes (Fig. 6C). According to Masson staining results, the Control group and GT group showed a smaller amount of newly formed collagen with disordered arrangement. The CGT group and CGT + TSPCs group had more newly formed collagen, and the collagen in the CGT + TSPCs group was more neatly arranged than that in other groups, with an increased proportion of collagen area (Fig. 6D–E). Further observation by transmission electron microscopy (Fig. 6F) revealed local collagen defects and disordered arrangement in the Control group and GT group. In contrast, more collagen was formed at the injury site in the CGT group and CGT + TSPCs group, with orderly arrangement of collagen fibers, which was consistent with the results of HE staining and Masson staining. In addition, Micro-CT scanning of rat Achilles tendons at 8 weeks postoperatively showed varying degrees of heterotopic ossification (HO) in all groups. The Control group and GT group exhibited more obvious heterotopic ossification at the injury site, while the CGT group and CGT + TSPCs group showed a significant partial inhibitory effect on heterotopic ossification (Fig. 6G). Compared with the Control group and GT group, the CGT group and CGT + TSPCs group significantly reduced the bone volume of traumatic heterotopic ossification in tendons (Fig. 6H), confirming that CGT partially inhibits the progression of HO.

To further assess the biomechanical and functional recovery of Sprague-Dawley rats following Achilles tendon injury, gait analysis was conducted utilizing the CatWalk XT system (Noldus, Netherlands). This comprehensive analysis encompassed various gait parameters, including spatiotemporal parameters (such as stride length, swing time, and swing speed) and intensity parameters, which serve as indirect indicators of the pain condition in the affected limb of the rats (Fig. S6A–B). The results revealed that the stride length in the CGT + TSPCs group was significantly greater than that observed in the Control and GT groups, indicating enhanced mobility of the affected limb in the CGT + TSPCs group. However, no statistically significant differences were observed in stride time and swing speed across the groups (Fig. S6C–E). Additionally, the average intensity of ground contact by the affected limb was found to be superior in the CGT + TSPCs group compared to the Control group (Fig. S6F). Fig. S6G illustrates the paw prints of the affected limb in Sprague-Dawley rats across different groups at 8 weeks post-operation. The assessment of the Achilles tendon functional index (AFI) revealed that the AFI scores in the CGT + TSPCs and CGT groups were significantly superior to those in the GT and Control groups (Fig. S6H). This indicates a more pronounced recovery of Achilles tendon function in the former groups. In summary, the application of CGT and CGT + TSPCs treatments could partially enhance functional recovery following Achilles tendon injury.

**Fig. 7:**
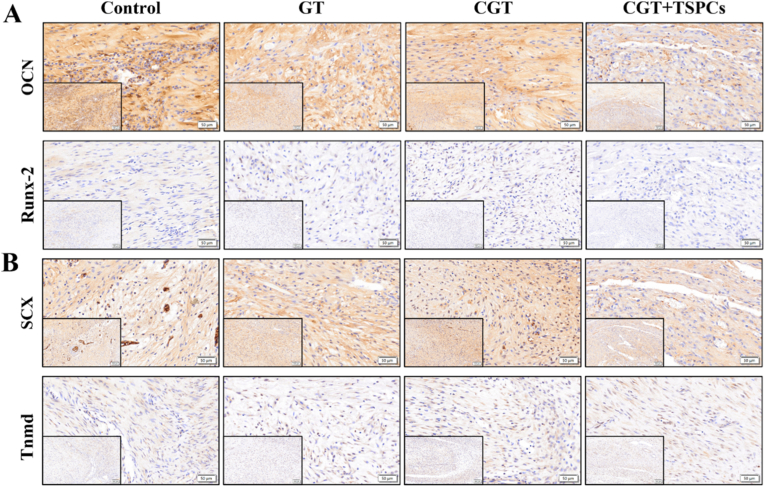
**Immunohistochemical staining for** (A) osteogenesis-related genes OCN and Runx-2, and (B)tenogenesis-related genes Scx and Tnmd, Scale bar: 50 μm and 100 μm (magnification).

In addition, we further detected the expression of tenogenesis-related genes and osteogenesis-related genes in the Achilles tendon tissues of SD rats in each group. Results of tissue immunostaining showed that the Control group and GT group contained a higher number of osteogenesis-related gene (OCN and Runx-2) positive cells, whereas the CGT group and CGT + TSPCs group had fewer OCN and Runx-2 positive cells. Compared with the CGT + TSPCs group, more OCN and Runx-2 positive cells were observed locally at the injury site in the CGT group (Fig. 7A, S7A-B). We simultaneously examined the expression of tenogenesis-related genes Scx and Tnmd, and the results revealed that Scx and Tnmd positive cells were distributed at the injury site in all groups. In comparison with the Control group and GT group, more Scx and Tnmd positive cells were observed around the injury site in the CGT group and CGT + TSPCs group (Fig. 7B–S7C-D). We also detected the expression level of IL-1β, and the results showed that the CGT group and CGT + TSPCs group partially inhibited the expression of IL-1β in the tendon tissue after injury (Fig. S2). These results demonstrate that CGT can partially inhibit the expression of osteogenesis-related genes and promote the expression of tenogenesis-related genes during tendon tissue repair. This indicates that CGT is beneficial for tendon injury repair, inhibits the overexpression of inflammatory factors in tendon tissue, and can partially prevent the occurrence of traumatic heterotopic ossification.

## 4 Discussion

In this study, we successfully constructed a ROS-responsive hydrogel loaded with capsaicin (CGT) and systematically verified its role in tendon injury repair. We confirmed that in a persistent inflammatory environment simulated by 10 ng/ml IL-1β for 3 days, CGT significantly ameliorated the dysfunction of TSPCs. On one hand, it promoted collagen fiber synthesis by upregulating the expression of tenogenic markers such as Scx, Tnmd, and Mkx, partially restoring the tenogenic differentiation capacity of TSPCs *in vitro*. On the other hand, it inhibited aberrant osteogenic differentiation of TSPCs by downregulating the expression of osteogenic markers. Mechanistically, CGT may reduce the secretion of proinflammatory factors such as IL-6 and TNF-α by inhibiting the PI3K-AKT-mTOR signaling axis, thereby improving the inflammatory microenvironment. *In vivo* experiments further confirmed that local injection of CGT combined with exogenous TSPCs promoted ordered collagen regeneration at the Achilles tendon injury site in rats, reduced inflammatory cell infiltration, significantly inhibited the occurrence of traumatic heterotopic ossification, and exhibited favorable biosafety.

Regulation of stem cell function has increasingly attracted attention in the field of tendon injury repair [[33], [34], [35], [36]]. As a stem cell population with self-renewal and multipotent differentiation potential in tendon tissue, TSPCs play a crucial role in tendon injury repair and tissue homeostasis [37,38]. Under normal physiological conditions, TSPCs can differentiate into tenocytes, synthesize and secrete collagen fibers, and promote the regeneration and functional reconstruction of tendon structures. However, the drastic changes in the local microenvironment after tendon injury severely impair the biological functions of TSPCs. Previous studies have reported that persistent low-grade inflammatory responses after tendon injury can induce TSPCs to produce large amounts of reactive oxygen species (ROS), which disrupt their differentiation balance by activating oxidative stress signaling pathways [39], leading to TSPCs differentiation imbalance, which may be the key cellular basis for tendon repair failure [11]. In this study, IL-1β intervention resulted in decreased tenogenic differentiation capacity and aberrantly enhanced osteogenic differentiation of TSPCs, verifying that inflammation can induce TSPCs differentiation imbalance. The differentiation fate of TSPCs directly determines the nature of repaired tissue: dominant tenogenic differentiation promotes functional tendon regeneration, while enhanced osteogenic differentiation leads to complications such as heterotopic ossification [40]. CGT maintains the dominance of tenogenic differentiation by protecting TSPCs from excessive inflammatory damage. Furthermore, the reparative efficacy observed in the CGT + TSPCs group surpassed that of the CGT alone group, indicating that the addition of exogenous TSPCs can significantly enhance repair efficiency. This enhancement may be attributed to the ability of CGT to create an optimal growth microenvironment for exogenous TSPCs, allowing TSPCs to perform tenogenic differentiation more effectively under the influence of CGT, thereby establishing a synergistic interaction. Future research should incorporate live-cell tracking technology to improve the evaluation of the long-term retention, stability, and viability of TSPCs following implantation. Specifically, lentiviral vectors with luciferase or fluorescent reporter genes can label TSPCs prior to implantation, enabling dynamic *in vivo* imaging to track TSPC survival, retention, and migration over time. This method clarifies the link between TSPC survival and tendon repair efficacy, and supports the synergistic repair mechanism of CGT and TSPCs. Additionally, no significant damage to major organs was detected post-hydrogel implantation across all groups, thereby affirming the favorable biosafety profile of CGT and suggesting its potential for clinical application.

As the core bioactive component in this study, capsaicin exerts multiple roles in the repair function of CGT. *In vitro* study showed that capsaicin can improve the inflammatory microenvironment by inhibiting the expression of proinflammatory factors, while directly regulating the differentiation direction of TSPCs to promote tenogenesis and inhibit osteogenesis. This is consistent with the previously reported biological activities of capsaicin, such as anti-inflammatory effects and regulation of stem cell functions [41,42]. As a TRPV1 agonist, the role of capsaicin in neurogenic inflammation and tendon healing has been documented. Capsaicin facilitates axon growth by activating TRPV1 receptors, a mechanism that involves calcium ion influx and the activation of protein kinase A (PKA) [43]. Within the central nervous system, capsaicin demonstrates antioxidant and anti-inflammatory properties through TRPV1 receptor activation, thereby mitigating the transmission of immunological oxidative and inflammatory signals to the brain. This action reduces the risk of brain damage associated with ischemia or excitotoxicity [44]. Additionally, capsaicin stimulates the release of neuropeptides such as calcitonin gene-related peptide (CGRP) and substance P (SP), which are integral to the inflammatory response [45]. Research conducted by Daniel et al. [20] identified a correlation between post-capsaicin treatment levels of substance P and the biomechanical properties of tendon repair. This suggests that capsaicin may enhance tendon healing by modulating neurotransmitter release. Additionally, another study reported that while capsaicin induces pain and hyperalgesia, its application to tendon tissue does not result in significant structural damage, thereby supporting its safety for use in clinical pain management [22]. Consequently, the role of capsaicin in tendon physiology may involve the modulation of neural activity and inflammatory processes. In this study, the introduction of TSPBA endows the biomaterial with potential ROS-responsive advantages. After tendon injury, inflammatory cell infiltration and tissue ischemia lead to elevated local ROS levels; excessive ROS not only exacerbate inflammatory responses but also directly damage TSPCs and surrounding tissues [39,46]. ROS-responsive hydrogels can accelerate degradation and release loaded drugs in a high-ROS microenvironment, achieving precise drug delivery at the injury site [47,48]. Notably, the effect of capsaicin depends on a reasonable delivery method. The GelMa@TSPBA hydrogel constructed in this study provides an ideal sustained-release carrier for capsaicin; the sustained-release property of the hydrogel enables capsaicin to maintain effective concentrations at the injury site, prolong its action time, and reduce the risk of systemic toxicity.

To further explore the molecular mechanism underlying the function of CGT, this study reported through transcriptome sequencing that the PI3K-AKT-mTOR signaling axis may be the key pathway through which CGT regulates inflammation and TSPC functions. As a classic intracellular signal transduction network, the PI3K-AKT-mTOR pathway is involved in regulating various biological processes such as cell proliferation, differentiation, and inflammatory responses [[49], [50], [51]]. In the tendon injury model, IL-1β activated this pathway, leading to increased phosphorylation levels of AKT and mTOR, thereby promoting the secretion of proinflammatory factors and the expression of osteogenic differentiation-related genes. CGT intervention reduced the expression of p-AKT and p-mTOR, decreased the release of proinflammatory factors such as IL-6 and TNF-α by inhibiting the transmission of inflammatory signals, and improved the tenogenic differentiation function of TSPCs. In this study, we conducted a preliminary investigation into the role of the PI3K-AKT-mTOR signaling axis in regulating the functions of TSPCs using a composite CGT. However, we did not compare its effects with those of other phosphorylation inhibitors targeting the PI3K-AKT signaling pathway, such as LY294002, or the mTOR inhibitor rapamycin. This limitation highlights the need for further in-depth exploration in future research. Notably, KEGG enrichment analysis showed that CGT also affects other signaling pathways such as MAPK and p53, suggesting that it may exert its functions through multi-pathway synergism. Wu et al. [52] reported that Geraniol can improve osteoarthritis by downregulating PI3K-AKT and MAPK signaling. The PI3K-AKT axis and MAPK are involved in regulating the maturation of cardiomyocytes [53]. These studies indicate that there is crosstalk between the PI3K-AKT-mTOR signaling pathway and the MAPK pathway, which requires further evaluation in future study. Moreover, angiogenesis is integral to the process of tendon repair [[54], [55], [56]]. The result of GSEA revealed alterations in genes associated with angiogenesis. Consequently, future research should explore the impact of capsaicin on angiogenesis in the context of tendon tissue injury.

This study has several limitations. *In vitro* study did not further explore the specific molecular mechanism by which capsaicin regulates the PI3K-AKT-mTOR pathway. The observation time of animal experiments was only 8 weeks postoperatively, failing to evaluate the long-term repair effect and biomechanical properties of CGT on tendons. Furthermore, while the effects of CGT in promoting tendon repair, as confirmed in this study, highlight the advantages of the composite system based on existing data, they do not entirely exclude the independent contributions of the hydrogel matrix's delivery-enhancing properties and capsaicin's intrinsic pharmacological effects. Additionally, the specific contribution of TSPBA-mediated ROS responsiveness to these effects remains unclear. Moreover, this study did not compare the efficacy of capsaicin with that of other anti-inflammatory drugs in the context of tendon injury repair. Future research should consider chemical modifications to enhance the ROS reactivity of the material and improve the sustained-release efficiency of the drug. By employing proteomic or metabolomic techniques, the synergistic mechanism between capsaicin and the hydrogel matrix can be elucidated, while also verifying the efficacy of non-ROS-responsive hydrogels. Concurrently, experiments using large animal models and long-term safety evaluations should be conducted to establish a foundation for clinical translation.

## 5 Conclusion

In summary, this study constructed a ROS-responsive hydrogel loaded with capsaicin, which alleviated the impairment of TSPC tenogenic function induced by IL-1β by inhibiting the phosphorylation of the PI3K-AKT-mTOR signaling axis. Further *in vivo* experiments confirmed that local injection of CGT loaded with exogenous TSPCs at the Achilles tendon injury site achieved the effects of promoting tendon regeneration and inhibiting heterotopic ossification. This study provides new insights into the design of functional biomaterials for tendon injury repair, but its long-term repair effect and specific clinical translation protocols require further exploration.

## CRediT authorship contribution statement

**Yun-Liang Zhu:** Writing – original draft, Software, Resources, Methodology, Data curation. **Si-Chao Gu:** Data curation. **Bao-Liang Lu:** Data curation. **Hu Sun:** Formal analysis. **Zai-Yong Guan:** Formal analysis. **Rui-Hua Zhou:** Validation, Supervision. **Ting-Yong Sun:** Visualization, Validation, Methodology. **Wen Gao:** Methodology, Investigation, Funding acquisition. **Shi-Yuan Fang:** Writing – review & editing, Writing – original draft.

## Ethics approval and consent to participate

The animal experiments were approved by the Animal Care Committee of the First Affiliated Hospital of University of Science and Technology of China (202304152353000471629).

## Human ethics

Not applicable.

## Consent to publication

Not applicable.

## Availability of supporting data

The data that support the findings of this study are available from the corresponding authors upon reasonable request.

## Funding

This work was supported by the 10.13039/100008696Zhejiang Provincial Health Science and Technology Program Project[2022KY1344].

## Declaration of competing interest

The authors declare that they have no known competing financial interests or personal relationships that could have appeared to influence the work reported in this paper.

## Data Availability

Data will be made available on request.
