# Supplementary material for: ROS-responsive hydrogel loaded with capsaicin promotes tenogenic differentiation of tendon stem/progenitor cells and enhances tendon injury repair
Source: Mater Today Bio. 2025 Dec 24;36:102707. doi: 10.1016/j.mtbio.2025.102707 (PMC12813339; doi:10.1016/j.mtbio.2025.102707)
Supplement: Multimedia component 1 [file mmc1.docx]

- ***Title page***

**ROS-responsive Hydrogel Loaded with Capsaicin Promotes Tenogenic Differentiation of Tendon Stem/Progenitor Cells and Enhances Tendon Injury Repair**

Yun-Liang Zhu^1,2 #^, Si-Chao Gu^2,3, #^, Bao-Liang Lu^2 #^, Hu Sun^2^, Zai-Yong Guan^1,^, Rui-Hua Zhou^1^ Ting-Yong Sun^1^, Wen-Gao^1*^, Shi-Yuan Fang^2*^

^1^ Department of Orthopedics, Gaoyou People's Hospital, The Third Clinical Medical College of Yangzhou University, Yangzhou, Jiangsu 225600, China

^2^ Department of Orthopedics, The First Affiliated Hospital of USTC, Division of Life Sciences and Medicine, University of Science and Technology of China, Hefei, Anhui,China

^3^ The Second Affiliated Hospital of Fuyang Normal University, Fuyang, Anhui 236001, China

^#^ These authors contributed equally to this work.

***Corresponding author**: Shi-Yuan Fang (Email: fangshiyuan2008@126.com), Department of Orthopedics, The First Affiliated Hospital of USTC, Division of Life Sciences and Medicine, University of Science and Technology of China, Hefei, Anhui,China

Wen-Gao (Email: szxgk2@163.com), Department of Orthopedics, Gaoyou People's Hospital, The Third Clinical Medical College of Yangzhou University, Yangzhou, Jiangsu 225600, China

**Supplementary Table**

**Table 1. WB antibodies used in this study**

|  | Antibody | Company | Cat# | Dilution | Country |
| --- | --- | --- | --- | --- | --- |
| Primary antibodies | Phospho-PI3 Kinase p110 beta (Ser1070) Polyclonal Antibody | ThermoFisher | BS-6417R | 1:1000 | USA |
|  | PI3 Kinase p110 Beta Polyclonal antibody | Proteintech | 20584-1-AP | 1:500 | China |
|  | Phospho-AKT (Ser473) Monoclonal antibody | Proteintech | 66444-1-lg | 1:2000 | China |
|  | AKT Polyclonal antibody | Proteintech | 10176-2-AP | 1:5000 | China |
|  | mTOR Rabbit pAb | Abclonal | A2445 | 1:1000 | China |
|  | p-mTOR Rabbit pAb | Abclonal | AP0094 | 1:500 | China |
|  | β-actin Rabbit pAb | Abclonal | AC006 | 1:10000 | China |
| Second antibodies | HRP-conjugated Goat Anti-Rabbit IgG(H+L) | Proteintech | SA00001-2 | 1:1000 | China |
|  | HRP-conjugated Goat Anti-Mouse IgG(H+L) | Proteintech | SA00001-1 | 1:1000 | China |

**Table 2. IF antibodies used in this study**

|  | Antibody | Company | Cat# | Dilution | Country |
| --- | --- | --- | --- | --- | --- |
| Primary antibodies | TNMD Rabbit pAb | Bioss | bs-7525R | 1:100 | China |
|  | Scleraxis Antibody (A-7) | SANTA CRUZ | sc-518082 | 1:200 | China |
|  | RUNX2 Polyclonal antibody | Proteintech | 20700-1-AP | 1:200 | China |
|  | Anti-Osteocalcin Mouse mAb | Servicebio | GB120012-50 | 1:500 | China |
| Second antibodies | Multi-rAb® CoraLite® Plus 594-Goat Anti-Rabbit Recombinant Secondary Antibody (H+L) | Proteintech | RGAR004 | 1:1000 | China |
|  | Multi-rAb™ CoraLite® Plus 594-Goat Anti-Mouse Recombinant Secondary Antibody (H+L) | Proteintech | RGAM004 | 1:1000 | China |
|  | Multi-rAb® CoraLite® Plus 488-Goat Anti-Rabbit Recombinant Secondary Antibody (H+L) | Proteintech | RGAR002 | 1:1000 | China |
|  | Multi-rAb® CoraLite® Plus 488-Goat Anti-Mouse Recombinant Secondary Antibody (H+L) | Proteintech | RGAM002 | 1:1000 | China |

**Table 3. Gene primers used in this study**

| Gene | Forward primers | Reverse primers |
| --- | --- | --- |
| Scx | 5'-AGAACACCCAGCCCAAACA-3' | 5'-CGGTCTTTGCTCAACTTTCT-3' |
| Tnmd | 5'-GTCACATTCTAAATGCAGAAG-3' | 5'-CTCCCCCAAAACAGGACAAT-3' |
| Mkx | 5'-CTATCGCCACAGGTAAGCCCA-3' | 5'-CCCACGTATCAGTTTCTCCCA-3' |
| IL-1β | 5'-CCCAAGCACCTTCTTTTCCTT-3' | 5'-TCAGACAGCACGAGGCATTT-3' |
| IL-6 | 5'-CTGATTGTATGAACAGCGATGATG-3' | 5'-GGTAGAAACGGAACTCCAGAAGAC-3' |
| TNF-α | 5'-CAAGAGCCCTTGCCCTAAGG-3' | 5'-CGGACTCCGTGATGTCTAAGTACTT-3' |

**Supplementary Figures**

**
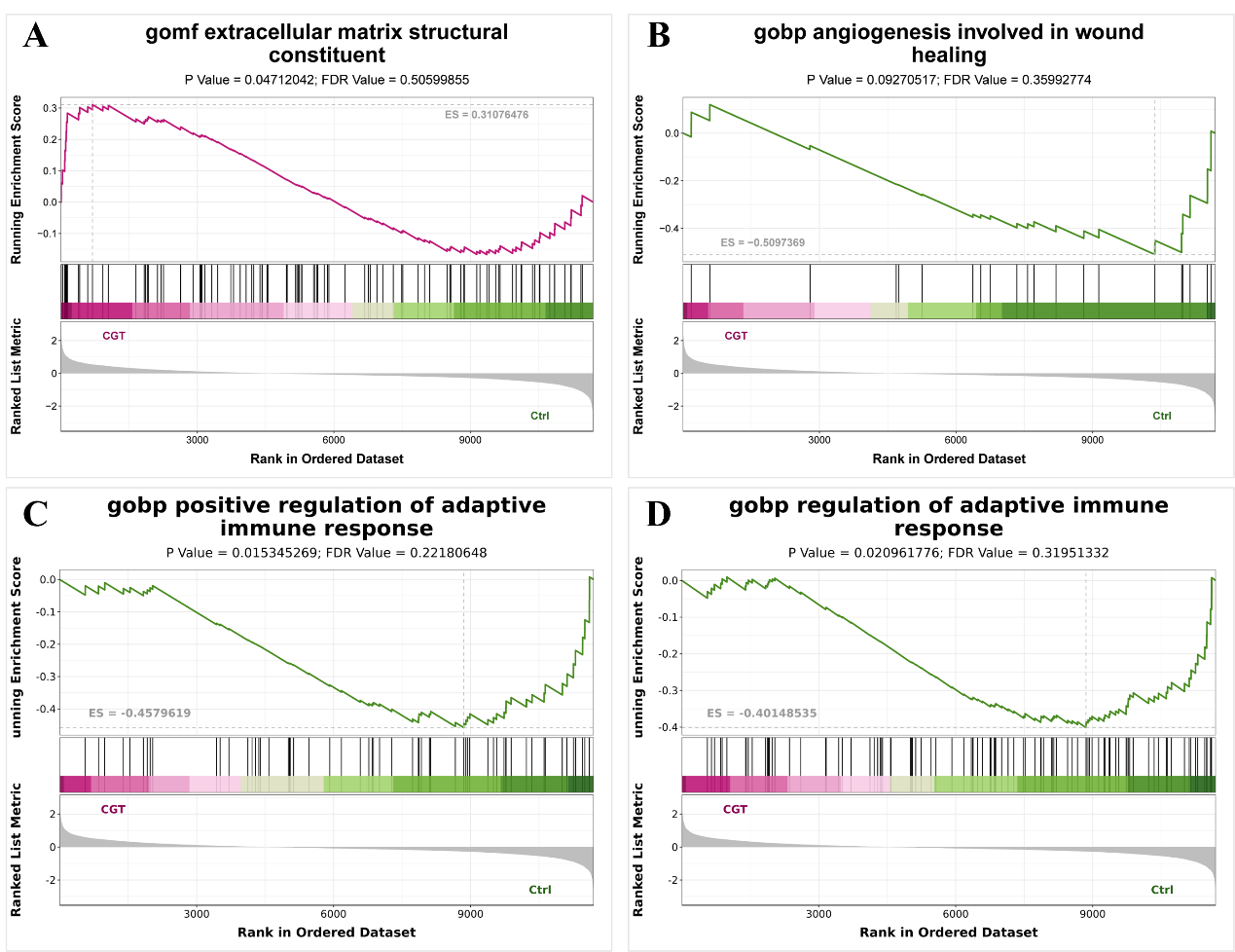
**

**Figure S1** GSEA plots associated with (A)extracellular matrix structural constituent, (B)angiogenesis involved in wound healing, (C)positive regulation of adaptive immune response, and (D)regulation of adaptive immune response.


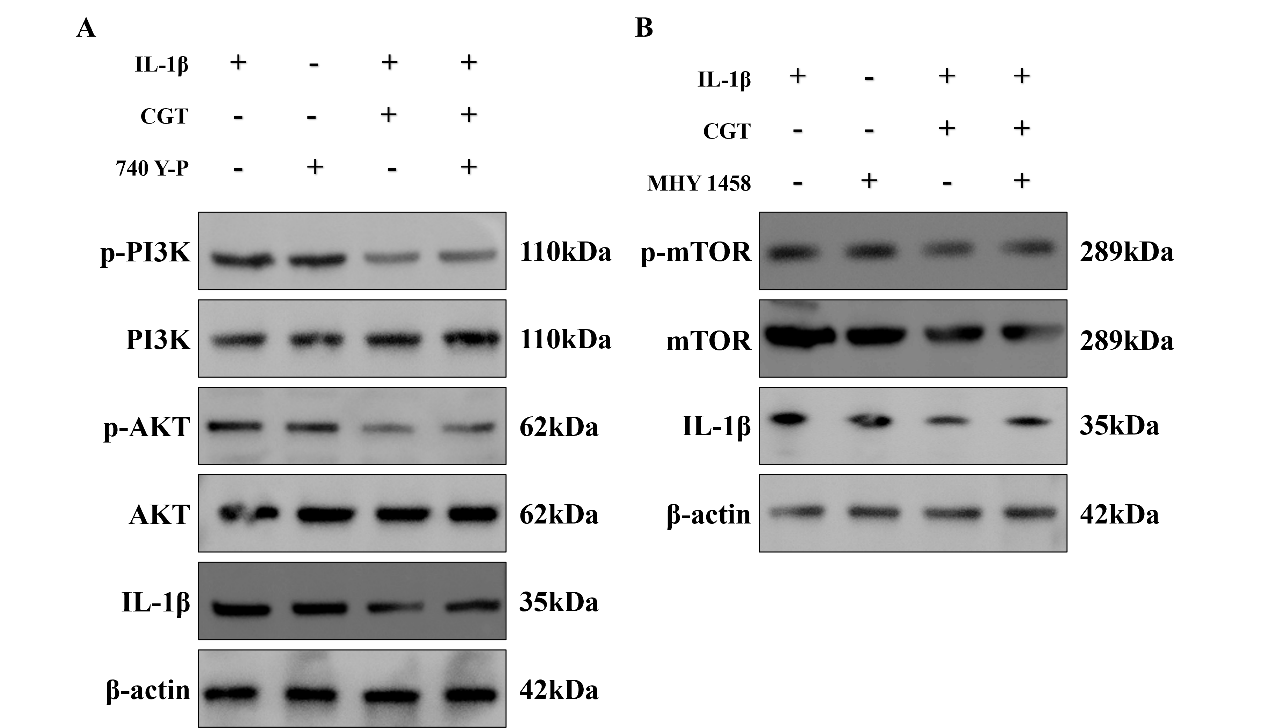


**Figure S2** Western blot (WB) results of protein expression of PI3K-AKT-mTOR signaling pathway related genes (AKT, p-AKT, PI3K, p-PI3K, mTOR, and p-mTOR) and IL-1β in different groups.


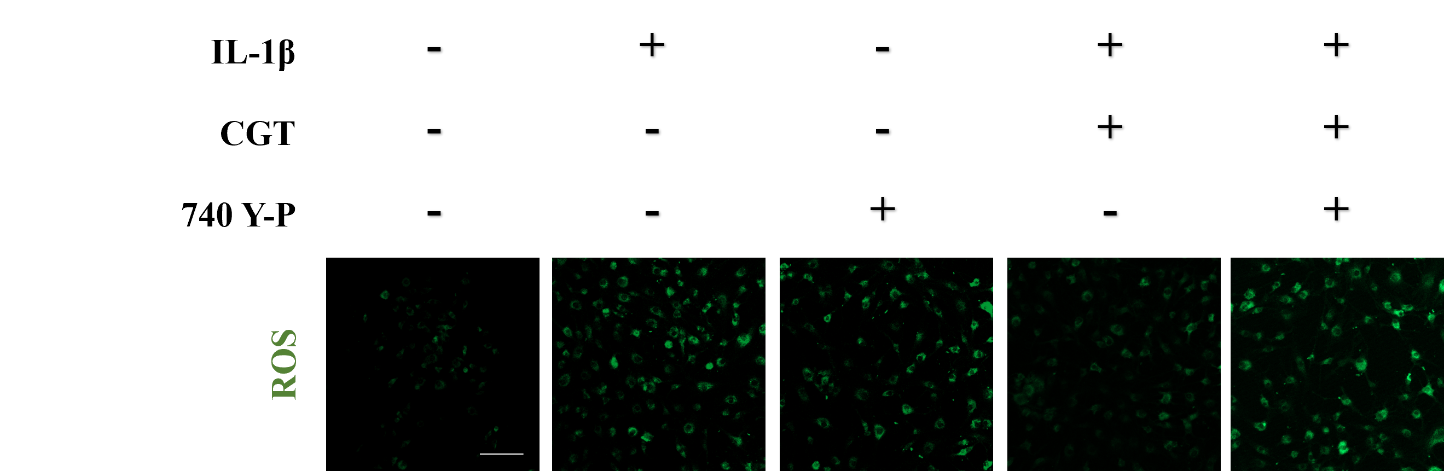


**Figure S3** Fluorescence analysis of intracellular ROS in different group. Scar bar:100 μm


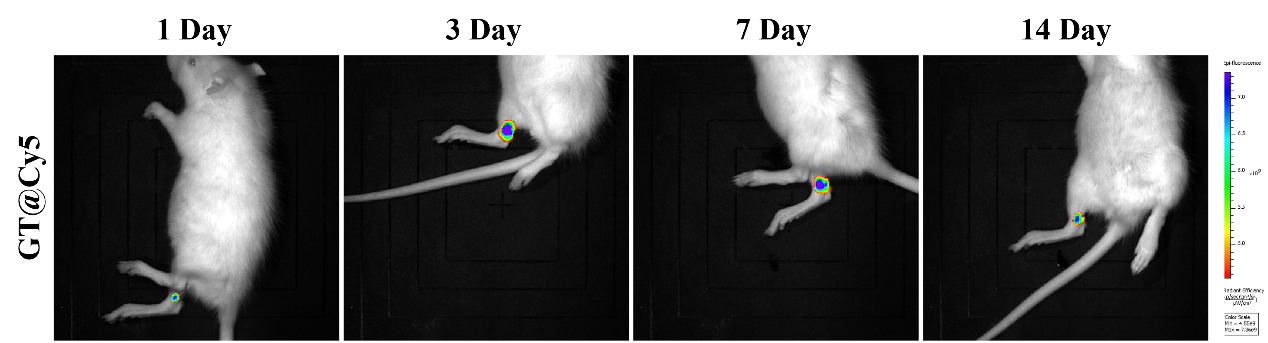


**Figure S4** Result of animal imaging techniques to detect GT@Cy5 release at different time points *in vivo*.


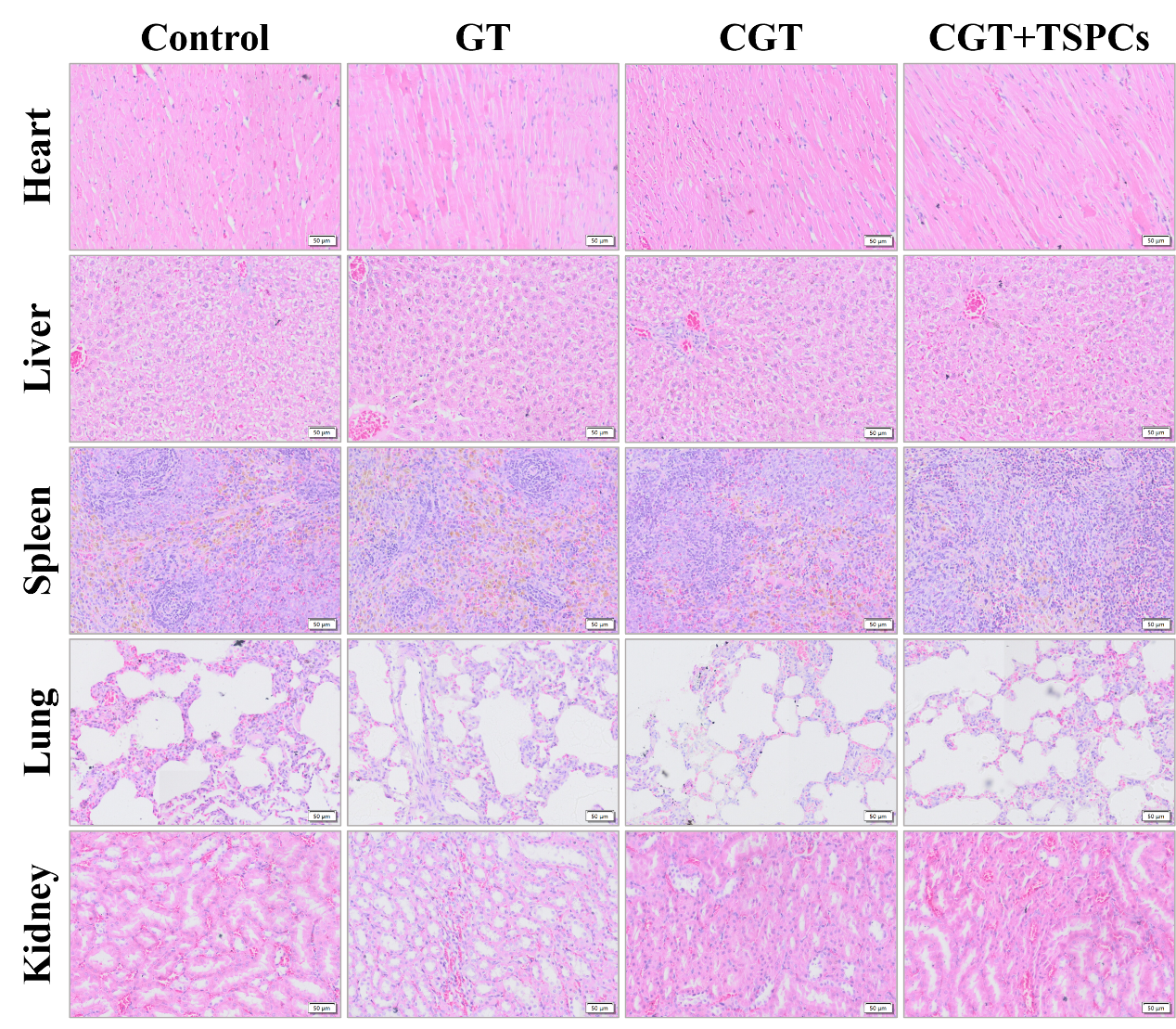


**Figure S5** H&E staining of vital organs in SD rat Achilles tendon injury model at 8 weeks after CGT treatment


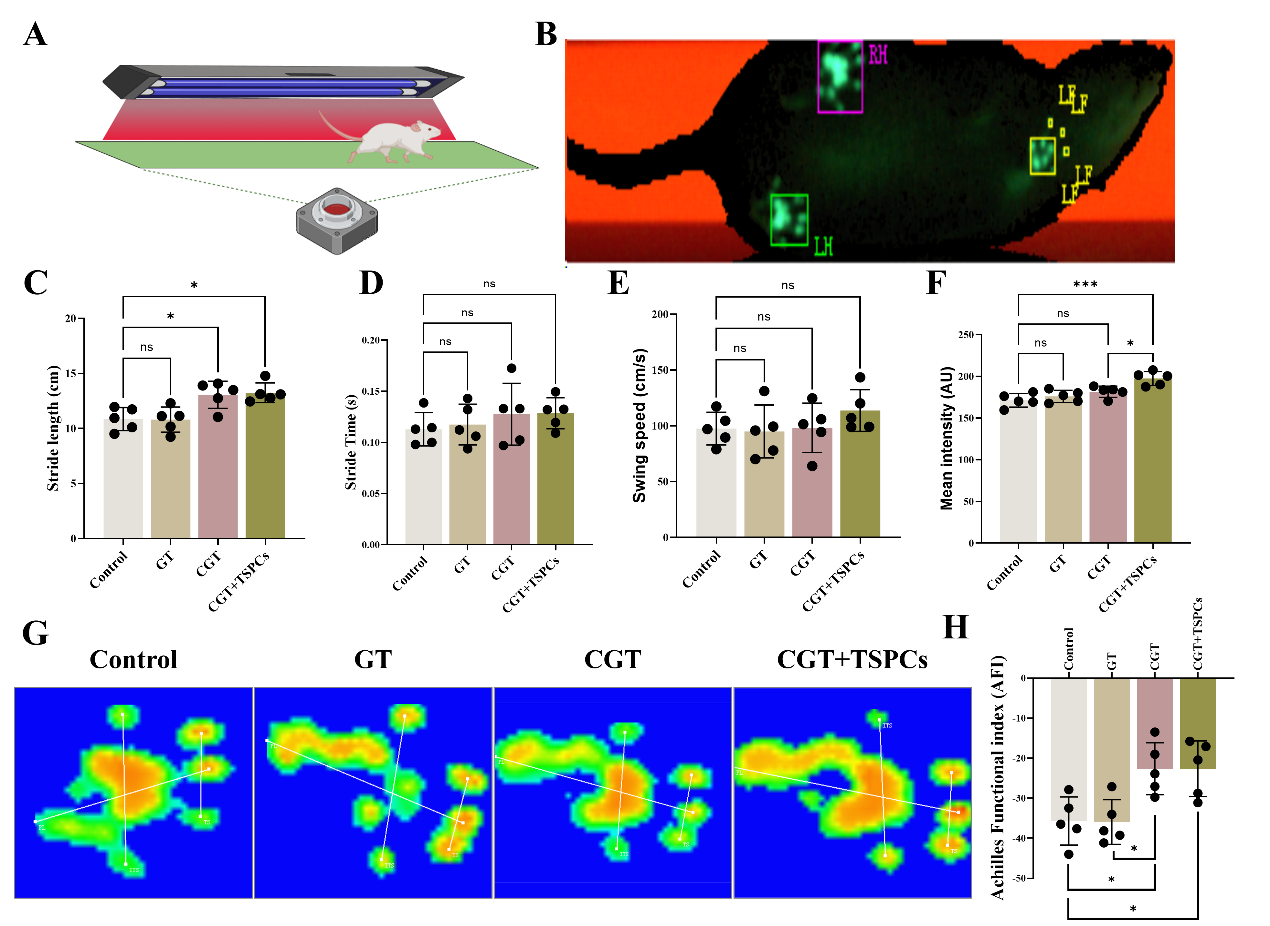


**Figure S6 Gait Analysis.** (A–B) Schematic diagrams of the gait analysis pattern; spatiotemporal parameter analysis including (C) stride length, (D) swing time, and (E) swing speed. (F) Average intensity of the affected limb’s contact with the ground. (G) Paw prints of the affected limb after surgery and (H) Achilles tendon function index. ns: not significant; **P* < 0.05, ****P* < 0.001.


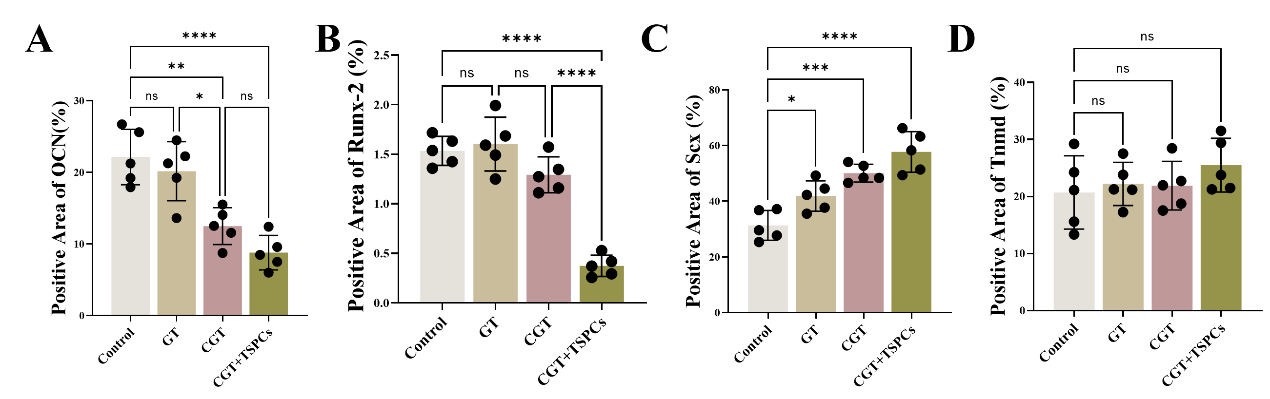


**Figure S7** Quantitative analysis of IHC staining for OCN, Runx-2, Scx, and Tnmd at 8 weeks after injury. **P*＜0.05, ***P*＜0.01, ****P*＜0.001, *****P*＜0.0001.


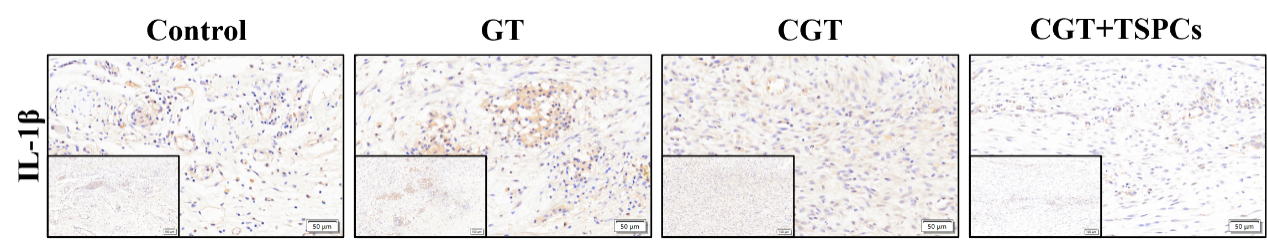


**Figure S8** Immunohistochemical staining of IL-1β in tendon tissues from the SD rat Achilles tendon injury model at 8 weeks after CGT treatment.
